# LONG-TERM CARE RESIDENT PERSPECTIVES ON PERSON-CENTERED CARE: INTERSECTIONS OF PRACTICE AND THEORY

**DOI:** 10.1093/geroni/igac059.2363

**Published:** 2022-12-20

**Authors:** Mary Lou Ciolfi, Catherine Taylor, Tracy Ericson, David Wihry, Jennifer Crittenden, Angela Hunt, Lenard Kaye, Paul Nebenzahl

**Affiliations:** University of Maine Center on Aging, Bangor, Maine, United States; University of Maine, Bangor, Maine, United States; The Cedars, Portland, Maine, United States; University of Maine, Orono, Maine, United States; UMaine Center on Aging, Bangor, Maine, United States; The Cedars, Portland, Maine, United States; University of Maine, Bangor, Maine, United States; The Mayer-Rothschild Foundation, Chicago, Illinois, United States

## Abstract

The Designation of Excellence in Person-Centered Long-Term Care is a multi-year study to redefine standards of long-term care delivery according to the preferences of residents, family, staff, and leaders. During 2021, as one component of the study, 247 residents from 23 communities across the country engaged in a participatory message board activity inquiring about their lived experiences of receiving care and services in their long-term care communities. Residents commented on their personal care, personal and shared spaces; family, friends, and community; and wishes and feelings. Using a phenomenological approach to data analysis to better understand the universal and the unique aspects of the experience of living in long-term care, key themes centered on the importance of staff (1) knowing, responding, and being attentive to the details of residents’ life history, care needs and preferences; (2) treating residents respectfully and as mature adults; and (3) demonstrating care and connection in resident interactions. These key themes and their related sub-themes intersect with several social science theories that underscore those factors promoting adaptability to changed environments while trying to preserve identity, belief systems, and values. Applying a conceptual lens to resident comments suggests programmatic strategies that long-term care providers can prioritize for promoting and operationalizing person-centered care. The poster will present a graphic representation of the intersections of person-centered care and existing theoretical frameworks using resident remarks to highlight central tenets of continuity theory, person-environment fit theory, and social identity theory.

